# LncRNA *CANT1* suppresses retinoblastoma progression by repellinghistone methyltransferase in *PI3Kγ* promoter

**DOI:** 10.1038/s41419-020-2524-y

**Published:** 2020-05-04

**Authors:** Hongyan Ni, Peiwei Chai, Jie Yu, Yue Xing, Shaoyun Wang, Jiayan Fan, Shengfang Ge, Yefei Wang, Renbing Jia, Xianqun Fan

**Affiliations:** 1 Department of Ophthalmology, Ninth People’s Hospital, Shanghai JiaoTong University School of Medicine, Shanghai, China 200011; 2 Shanghai Key Laboratory of Orbital Diseases and Ocular Oncology, Shanghai, China 200011

**Keywords:** Paediatric cancer, DNA methylation

## Abstract

Retinoblastoma (RB) is the most common malignant intraocular tumor of childhood. Recent studies have shown that long noncoding RNAs (lncRNAs), which are longer than 200 bp and without protein-coding ability, are key regulators of tumorigenesis. However, the role of lncRNAs in retinoblastoma remains to be elucidated. In this study, we found that the expression of lncRNA *CASC15-New-Transcript 1* (*CANT1*) was significantly downregulated in RB. Notably, overexpression of *CANT1* significantly inhibited RB growth both in vitro and in vivo. Furthermore, lncRNA *CANT1*, which was mainly located in the nucleus, occupied the promoter of phosphoinositide 3-kinase gamma (*PI3Kγ*) and blocked histone methyltransferase hSET1 from binding to the *PI3Kγ* promoter, thus abolishing hSET1-mediated histone H3K4 trimethylation of the *PI3Kγ* promoter and inhibiting *PI3Kγ* expression. Furthermore, we found that silencing *PI3Kγ* either by lncRNA *CANT1* overexpression or by *PI3Kγ* siRNA, reduced the activity of PI3K/Akt signaling and suppressed RB tumorigenesis. In summary, lncRNA *CANT1* acts as a suppressor of RB progression by blocking gene-specific histone methyltransferase recruitment. These findings outline a new *CANT1* modulation mechanism and provide an alternative option for the RB treatment.

## Introduction

Long noncoding RNAs (lncRNAs) are transcripts longer than 200 bp with no apparent protein-coding ability, that are involved in numerous important biological phenomena such as X chromosome inactivation, chromosome conformation shaping, and DNA damage repair^1–4^. The functional roles and mechanism of action of some classically defined lncRNAs are well understood. For instance, lncRNA *XIST* coats the X chromosome, is expressed only from the inactive X chromosome (Xi), and is essential for the silencing process^2,5,6^. LincRNA-*ROR* is an important factor for the reprogramming process because its depletion or overexpression results in reduced or increased efficiency of reprogramming fibroblasts to iPSCs^7^. LncRNA *non-coding RNA activated by DNA damage* (*NORAD*) interacts with proteins involved in DNA replication and repair in steady-state cells and localizes to the nucleus upon replication stress or DNA damage stimulation^8,9^. However, the functions of the majority of lncRNAs are unknown, and it is necessary to explore the functions of lncRNAs.

As lncRNAs play a key role in the maintenance of homeostasis, aberrant lncRNA expression may be an important trigger for a variety of diseases. LncRNA *BACE1*-*AS* levels have been found to be increased along with amyloid β levels across different regions in postmortem brains from Alzheimer’s disease patients, and *BACE1*-*AS* protects *BACE1* mRNA from degradation^10^. LncRNA *myocardial infarction associated transcript* (*MIAT*) is notably elevated in angiotensin II (ANG-II)-induced cardiac hypertrophy and forms a feedback loop with vascular endothelial growth factor and *miR-150-5p* to regulate endothelial cell function^11,12^. Therefore, aberrant lncRNA expression has attracted increasing attention in the study of the pathogenesis of the human disease.

Since many lncRNAs epigenetically regulate their targets, which are either tumor suppressors or oncogenes, the lncRNAs are closely associated with tumorigenesis. We previously reported that lncRNA-*ROR* serves as an oncoRNA that regulates its target by blocking the binding of histone methyltransferase G9A to its target gene^13^. LncRNA *GAU1* overexpression in retinoblastoma cisactivates the expression of its target gene *GALNT8* to induce retinoblastoma tumorigenesis^14^. LncRNA *CANT1* is a novel tumor suppressor in uveal melanoma and activates a novel *CANT1-JPX/FTX-XIST* long noncoding pathway by directly binding to the promoters of lncRNAs *JPX* and *FTX* and restoring the histone H3K4 methylation of their promoters^15^. Thus, correcting lncRNA-guided abnormalities may become an attractive strategy for controlling human malignancies.

Retinoblastoma is an aggressive childhood malignancy of the developing retina that is initiated by the mutation of both *RB* alleles, thus leading to the functional loss of RB protein (pRB)^16,17^. Following the *RB* mutation in the retinoblastoma, other important factors, including genetic and epigenetic alterations, contribute to tumor formation^18^. It has been reported that pRB inactivation collaborates strongly with *MYCN* overexpression, giving rise to retinoblastoma in mice^19,20^. In addition, epigenetic alterations may drive retinoblastoma formation by inducing histone H3K4 trimethylation and H3K9/H3K14 acetylation of the spleen tyrosine kinase (*SYK*) oncogene promoter and activate its expression, which is required for tumor cell survival^21^. These findings reveal the mechanisms underlying rapid retinoblastoma progression following *RB1* inactivation and provide a basis for further investigation of new regulatory mechanisms and promising therapeutic approaches for RB tumor progression.

Here, we successfully identified that lncRNA *CANT1* functions as a noncoding RB suppressor. Using epigenetic approaches, we found that lncRNA *CANT1* acts as a necessary suppressor playing a vital regulatory role in RB tumorigenesis and we identified a novel type of histone modification that inhibits *PI3Kγ* transcription.

## Materials and methods

### Cell culture

Human 293T cells (obtained from ATCC) were cultured in DMEM (GIBCO, USA) and human retinal pigment epithelial cell line ARPE19 (obtained from ATCC) was cultured in DMEM/F12 medium (GIBCO, USA)^22,23^. The retinoblastoma cell lines Y79 (obtained from ATCC), Weri-Rb1 (obtained from ATCC), and RB44 (kindly provided by Heping Xu, Central South University, Changsha, China) were cultured in RPMI-1640 medium (GIBCO, USA)^24–26^. All media were supplemented with 10% fetal bovine serum (GIBCO, USA), 1% penicillin and streptomycin, and cells were incubated at 37 °C in a humidified 5% CO_2_ atmosphere.

### Bioinformatics analysis

LncRNA profiling data are available via the Gene Expression Omnibus (GEO): GSE111168^14^. Total RNA from lncRNA *CANT1*-knockdown RB and control RB cells was isolated and quantified. The concentration of each sample was measured by a NanoDrop 2000 (Thermo Scientific, USA). The quality was assessed by an Agilent2200 (Agilent, USA). The sequencing library of each RNA sample was prepared by using an Ion Proton Total RNA-Seq Kit v2 according to the protocol provided by the manufacturer (Life Technologies, USA).

### Real-time quantitative polymerase chain reaction (RT-qPCR)

Total RNA was extracted using TRIzol Reagent (ThermoFisher Scientific, USA) according to the manufacturer’s instructions. For the analysis of mRNA expression, cDNA synthesis was performed using the PrimeScript RT Reagent Kit with gDNA Eraser (Takara Biomedical Technology, Beijing, China). PCR analysis was performed using KlenTaq I mix, and amplified PCR products were quantified and normalized using *GAPDH* as a control. The PCR cycle parameters for lncRNA *CASC15* and lncRNA *CANT1* expression were as follows: 40 cycles of denaturation at 95 °C for 30 s, 65 °C for 30 s, extension at 72 °C for 30 s, and a final extension at 72 °C for 5 min. RT qPCR was performed using the SYBR Premix ExTaq (Takara Biomedical Technology, Beijing, China) under standard conditions according to the manufacturer’s instructions and also normalized using *GAPDH* as a control. The primers are listed in Supplementary Table S1.

### Plasmid construction and lentivirus packaging

The lncRNA *CANT1* plasmid was constructed as previously described^15^. The 293T cells were transfected using Lipofectamine 2000 reagent (Invitrogen, USA) with 3 μg CMV-*CANT1*, 3 μg pMD2.D, and 6.0 μg PsPax. The medium was replaced with 10 mL of fresh DMEM after 6 h. The virus-containing supernatants were collected at 48 and 72 h after transfection and then mixed and filtered through a 0.45 μm cellulose acetate filter (Sartorius, German). The viral supernatants were concentrated with Amicon Ultra-15 Centrifugal Filter Units (Millipore, USA) and spun at 5000 rpm for 30 min. Colonies were selected for subsequent culturing after incubation with 4 μg /mL puromycin for 4 weeks.

### Western blot analysis

Total protein was extracted using RIPA lysis buffer (Beyotime Biotechnology, Shanghai, China), and the protein concentration was determined using a BCA Kit (Beyotime Biotechnology, Shanghai, China). Equal amounts of protein were separated by sodium dodecyl sulfate-polyacrylamide gel electrophoresis (SDS-PAGE) and transferred to polyvinylidene difluoride membrane (Millipore, USA). The membrane was blocked with 5% nonfat milk for 1 h and incubated with primary antibodies overnight at 4 °C, followed by secondary antibodies for 1 h at room temperature. Protein bands were detected using a BIO-RAD imaging system (BIO-RAD, Hercules, CA, USA). The following antibodies were used: anti-PI3Kγ (1:1000 dilution; CST 5405S), anti-AKT (1:1000 dilution; CST 4691S), anti-phospho-AKT (Ser473) (1:2000; CST 4060S), and anti-GAPDH (1:10000 dilution; Sigma G9295).

### siRNA transfection

*PI3Kγ* and negative control siRNAs were designed and synthesized by Biomics (Shanghai, China). Y79 and Weri-Rb1 cells were transfected using Lipofectamine 2000 (Invitrogen, USA) according to the manufacturer’s protocol. Briefly, the cells were seeded at 2 × 10^5^ cells per well in 6-well plates and transfected with 125 pmol of siRNA (target gene or negative control) using Lipofectamine 2000 in Opti-MEM I Reduced Serum Medium (GIBCO, USA). After 48 h of transfection, the cells were harvested in TRIzol for RNA isolation or lysed in RIPA lysis buffer for Western blotting. Twenty-four hours after transfection, the cells were harvested by centrifugation and used for tumor assays.

### CCK8 cell viability assay

Cells were seeded at a density of 2000 cells per well in flat-bottomed 96-well plates. At the end of the incubation time, 10 μl of Cell Counting Kit-8 (CCK-8; Dojindo) solution was added to each well. After 4 h, the optical density at 450 nm was determined using a microplate reader (Varioskan Flash; Thermo, USA), and the absorbance values were normalized to the values of the cells at 0 h.

### Soft agar assay

A volume of 1 mL of 0.6% agar in the complete medium was spread in each well of a 6-well plate; 5000 cells were suspended in 1.0 mL of 0.3% agar complete medium and seeded into the upper layer. The cells were cultured with 300 µL of complete medium for 4 weeks. The colonies in soft agar were stained with 0.005% crystal violet and then photographed.

### Mouse xenograft experiments

All procedures were conducted in accordance with the ARVO Statement for the Use of Animals in Ophthalmic and Vision Research. All animals were cared for according to the guiding principles in the care and use of animals. The animal experiments were approved by the Animal Care and Use Committee at Shanghai JiaoTong University School of Medicine. All experiments conform to the guide for care and use of laboratory animals published by the National Insitutes of Health (NIH Publication 85-23, revised in 1996).

For xenograft experiments, 4-week-old male BALB/c nude mice were used. Mice were randomly divided into two groups: the Weri-Rb1 group (*N* = 7 eyes) and the Weri-Rb1-*CANT1* group (*N* = 7 eyes). The method for the inoculation of tumor cells into the posterior segments of the eye was as follows: nude mice were anaesthetized by intraperitoneal injection of a ketamine (final concentration: 10 mg/mL) and xylazine (final concentration: 1 mg/mL) mixture (0.01 mL/g mouse weight) and with alcaine ocular surface anesthesia. Under a surgical microscope, a sharp 30-gauge needle was used to make two holes through the sclera, one into the intravitreal space to reduce intraocular pressure and one tangentially through the sclera into the subretinal space for injection. RB cells (1 × 10^6^) were injected through the second hole into the choroid and subretinal space using a 1.5 cm, 33-gauge blunt end microinjection needle (7803-05, Hamilton, Reno, NV, USA). After the injection, eyes were covered with ophthalmic bacitracin ointment and buprenorphine was administered for relieving pain relief.

### Immunohistochemistry (IHC)

Tissues were embedded in paraffin, deparaffinized with xylene and rehydrated. Antigen retrieval was performed by heating in sodium citrate buffer (pH 6.0). The sections were blocked with 3% hydrogen peroxide for 20 min and then in 10% goat serum for 5 min. For tissue microarray immunohistochemical staining, tissues sections were incubated at 4 °C overnight with a rat anti-human PI3Kγ antibody (CST, USA) at a dilution of 1:100. Tissues were then rinsed in PBST (PBS containing 0.05% Triton X-100), and biotinylated anti-rat secondary antibody was added at a 1:500 dilution and incubated at room temperature for 1 h. After washing twice with PBST, the slides were incubated with streptavidin–horseradish peroxidase (BD Biosciences, USA) and diaminobenzidine substrate for colorimetric development.

### Cytoplasmic and nuclear RNA isolation

Cytoplasmic and nuclear RNA was extracted using Thermo Fisher BioReagents (Thermo Fisher, USA) according to the manufacturer’s instructions. RT-qPCR was performed to amplify the localized lncRNA *CANT1* as follows: 1 μL of 3× Klen-Taq I Mix, 1 μL of cDNA, and 0.5 μL of each 10 μM primer were combined under liquid wax. After incubation at 95 °C for 2 min, the cDNA was amplified with 40 cycles of 95 °C for 30 s, 65 °C for 30 s, and 72 °C for 30 s (extension), and with a final extension at 72 °C for 5 min.

### RNA Fish

RNA FISH was performed using a fluorescent in situ hybridization kit (RiboBio, China) following the manufacturer’s instructions. The lncRNA *CANT1* probes were designed and synthesized by the RiboBio Company. Briefly, cells were collected after transfection with the corresponding vector for 48 h and subsequently seeded on glass coverslips. Fluorescence detection was performed with a microscope (BX41; Olympus, Japan).

### Chromatin oligonucleotide precipitation (ChOP)

The ChOP assay was performed as previously described^15^. Cells were fixed using 1% formaldehyde (Sigma-Aldrich, USA) for 10 min at room temperature and centrifuged at 3000 rpm for 15 min. The pellet was suspended in 300 μL of buffer A (10 mM Tris-HCl, pH 7.4, 10 mM NaCl, 3 mM MgCl_2_, and 0.5% v/v NP-40) and incubated for 15 min on ice. The nuclei were harvested in 150 μL of buffer B (50 mM Tris-HCl, pH 7.9, 0.5 mM PMSF, 0.1% SDS, 10 mM EDTA,100 U/mL RNase, and protease inhibitors) and incubated on ice for 10 min. An equal volume of buffer C (15 mM Tris-HCl, pH 7.9, 1 mM EDTA, 1% Triton X-100, 150 mM NaCl, 0.5 mM PMSF, 100 U/mL RNase, and protease inhibitors) was added, and the samples were sonicated (10 s on, 15 s off, output 30%, 4 min). Sonicated DNA was found to be enriched in the range of 200–500 bp. After centrifugation, 150 μL aliquots of sonicated chromatin were combined with 100 pmol of either biotinylated antisense oligos against the target RNA or biotinylated scrambled oligos, incubated at an appropriate annealing temperature for 5 min and then slowly cooled to room temperature. A 50 μL volume of beads was used to capture the biotinylated DNA/RNA complexes for 25 min at room temperature with gentle rotation. After 3 washes, 150 μL of diethyl pyrocarbonate (DEPC) water was used for elution at 70 °C for 5 min. After crosslink reversal and purification, the samples were ready for PCR. A TaqMan assay using the ABI 7500 Real-Time PCR System was performed to detect the quality of the lncRNA *CANT1* pulled down by Dynabeads MyOne Streptavidin C1 beads. Primers and probes labeled at their 5′ and 3′ ends with FAM and minor groove binder or black hole quencher-1 were designed to target lncRNA *CANT1*. The amplification reactions were optimized individually for all of the probes and associated primers. Each reaction was conducted in a total volume of 10 μL consisting of 0.25 μL of 10 mM dinucleotide triphosphates (dNTPs), 0.1 μL of the TaqMan probe, 0.6 μL of 25 mM MgCl_2_, 2 μL of 5× Q buffer, 0.1 μL of 5 U/μL Hotstart, 0.1 μL of the reference ROX dye, 0.25 μL of each 10 μM primer, and 4 μL of the template.

### Chromatin immunoprecipitation (ChIP)

ChIP was performed using an EZ-Magna ChIP A/G kit (Millipore) according to the manufacturer’s instructions. Anti-hSET1 was purchased from Abcam and anti-H3K4me3 was purchased from Millipore. Anti-RNA polymerase-II (pol-II; Millipore) was used as a positive control antibody and normal mouse IgG was used as a negative control. See Supplementary Table S1 for a list of primers for ChIP-qPCR.

### Statistical analysis

Experiments were performed in triplicate when indicated, and the data were presented as the means ± SEM. The comparative threshold cycle method was applied in the RT-qPCR assay according to the ΔΔ threshold cycle method. The differences between two groups were analyzed using the unpaired two-sided Student’s *t* test. A *P* value <0.05 was considered statistically significant.

## Results

### *CANT1* lncRNA expression is downregulated in retinoblastoma

**Fig. 1:**
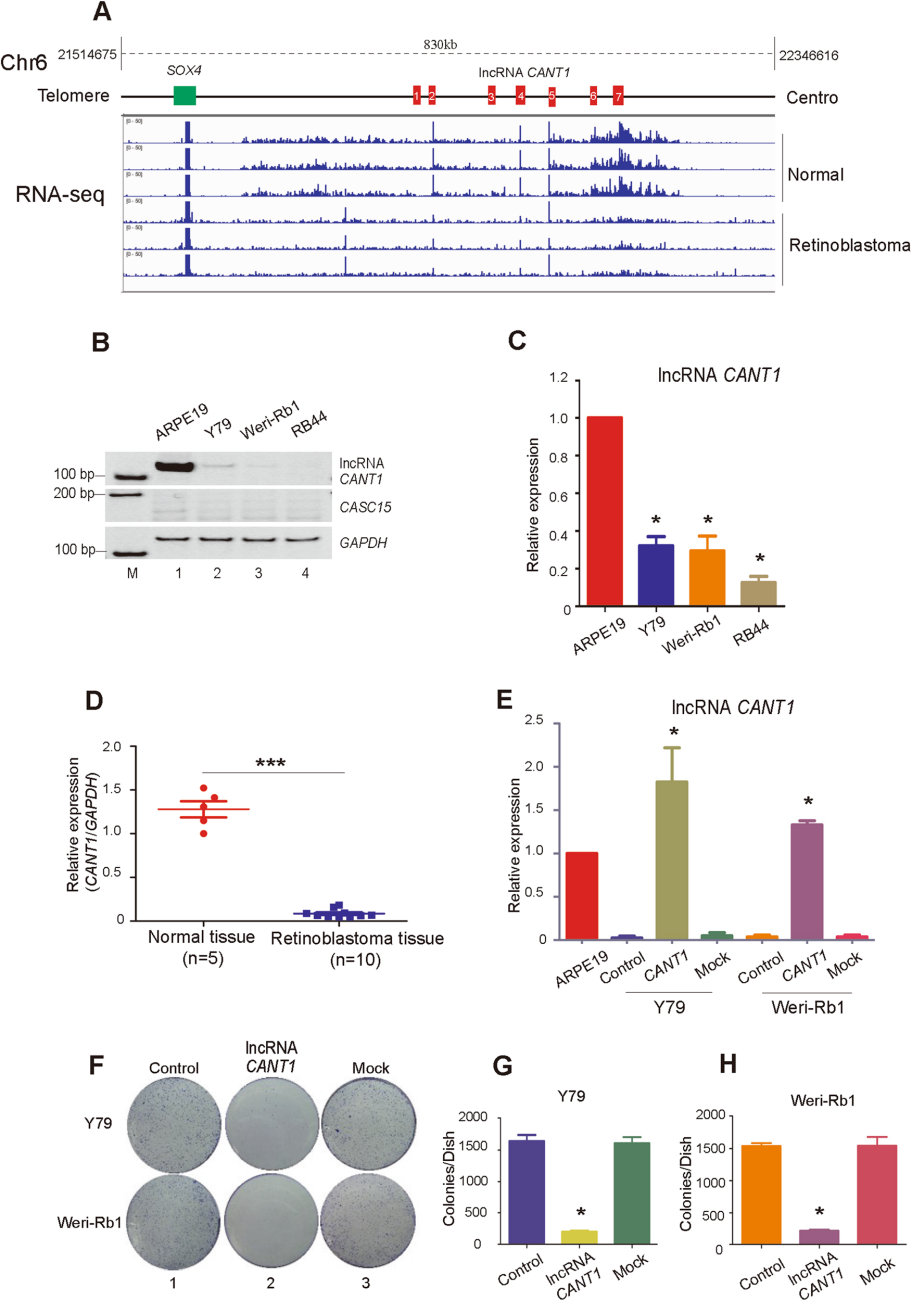
Identification of *CANT1* lncRNA. **a** RNA-sequence analysis was performed to evaluate the transcriptome of three retinoblastoma samples and normal retina samples. Red chart: the exons of lncRNA *CANT1*. Green chart: normal expression of the *SOX4* gene in Chr6p22.3. **b** Expression of *CANT1* and *CASC15* in three RB cell lines: Y79, Weri-Rb1, and RB44. ARPE19 cells were used as normal control. *GAPDH* was used as the internal control; M: marker. **c** Real-time PCR was performed to show *CANT1* expression level in RB cells; data are presented as the mean ± SEM. *P < 0.05 compared with ARPE19. **d** Real-time PCR examination of *CANT1* expression in RB tissue. Normal tissues were used as a control. The relative values were normalized to the GAPDH expression level and are presented as mean ± SEM. ***P < 0.001. **e** Real-time PCR showing overexpression of *CANT1* in RB cell lines Y79 and Weri-Rb1. Wild-type tumor cells were used as control. Mock, an empty vector. Data are presented as the mean ± SEM. *P < 0.05 compared with the respective control. **f** A colony formation assay was performed to assess the tumor growth of RB cells. **g**, **h** Colony numbers of Y79 (**g**) and Weri-Rb1 (**h**) cells were counted in three independent plates. All of the data are presented as the mean ± SEM. *P < 0.05: compared with the control.

A genome-wide RNA-sequencing analysis of three retinoblastoma tissues and paired normal tissues that we previously reported can be accessed via GEO: GSE111168^14^. The bioinformatics analysis showed that the level of *CASC15* lncRNA was lower in tumor tissues than that in normal tissues (Fig. 1a). We next identified which transcript existed on the chromosome 6p22.3 locus in RB. Different transcripts exist on the chromosome 6p22.3 locus in different tissues, for example, *CASC15* is 1902 bp in length with 12 exons according to databases in the University of California, Santa Cruz (UCSC) and National Center for Biotechnology Information (NCBI) databases, and lncRNA *CANT1* (GenBank: KP981381.1) is 1114 bp with 7 exons, which was confirmed by our previous research^15^. Therefore, we designed two isoform-specific primers to differentiate the transcripts (Supplementary Fig. S1A). The data showed that lncRNA *CANT1* expression was significantly decreased in RB cells compared with normal ARPE19 cells, however, *CASC15* was weakly expressed in both ARPE19 and RB cell lines (Fig. 1b). We also further confirmed *CANT1* expression by RT-PCR (Fig. 1c). To determine the clinical relevance of *CANT1* in RB, we collected human RB tissue samples (Table S2) to examine *CANT1* expression. The expression of *CANT1* was markedly reduced in RB tissues compared with normal tissues (Fig. 1d). These data show that lncRNA *CANT1* is alternatively spliced from chromosome 6p22.3 and is likely to play an unknown role in RB tumorigenesis.

To explore the potential role of *CANT1* in RB, we overexpressed lncRNA *CANT1* in RB cell lines. Therefore, we constructed a plasmid containing the full-length *CANT1* sequence and packaged it in a lentivirus for transfection into Y79 and Weri-Rb1. As expected, lncRNA *CANT1* was successfully overexpressed in Y79 and Weri-Rb1 cells (Fig. 1e).

### *CANT1* modulates RB tumorigenesis in vitro and in vivo

**Fig. 2:**
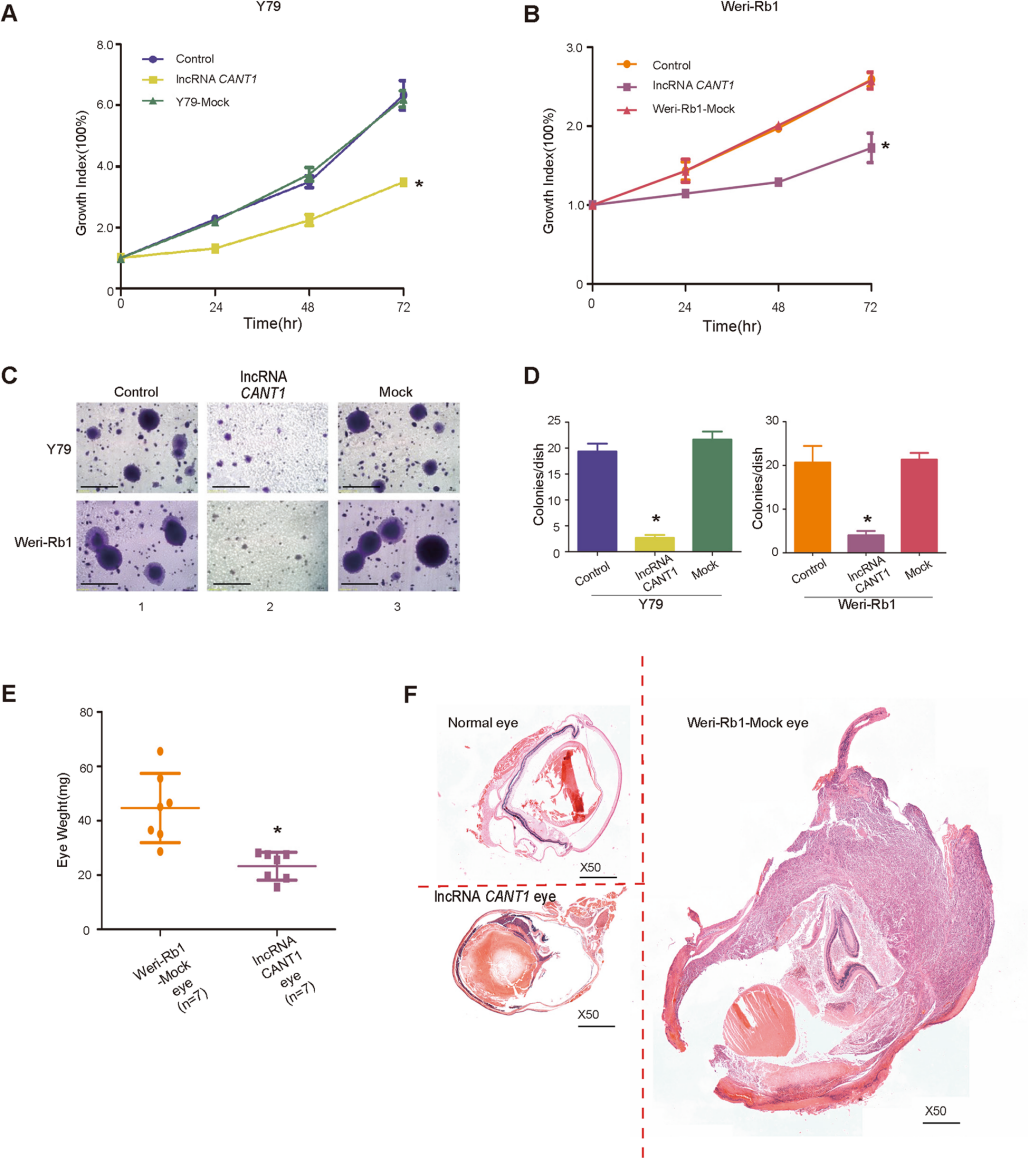
Functional roles of *CANT1* lncRNA in RB. **a**, **b** A CCK8 assay was performed to measure the cell growth rate of *CANT1*-overexpressing Y79 and Weri-Rb1 cells. The experiments were performed in triplicate, and the absorbance at day 1 was set as 100%. *P < 0.05 compared with the control and mock. **c**, **d** A soft agar assay was used to assess the tumor formation ability in vitro. **c** Small colonies were observed and counted under the microscope. **d** Colony count statistics showed a significant reduction in colonies formed by *CANT1*-overexpressing cells. Colony numbers were counted in three independent soft agar plates. All of the data are presented as the mean ± SEM. *P < 0.05 compared with the control and mock. Scale bar: 600 μm. **e**, **f** Eye weight of the orthotropic xenograft formed by Weri-Rb1 cells injected into the vitreous cavity with or without *CANT1* overexpression at 40 days after implantation; *n* = 7, *P < 0.05 compared with the mock. **f** Representative images of H&E staining for the evaluation of tumor formation in vivo. Scale bar: 200 μm.

We next investigated whether the RB tumor characteristics were altered after *CANT1* overexpression. In a colony formation assay, the number of colonies of *CANT1*-overexpressing Y79 cell (Fig. 1f upper and Fig. 1g) and Weri-Rb1 cell (Fig. 1f bottom and Fig. 1h) colonies was significantly reduced. In addition, we used a CCK8 assay to evaluate tumor cell growth. As expected, the RB cell growth rate was significantly lower than the wide-type RB cell growth rate (Fig. 2a, b). Next, we used a classical soft agar assay to examine tumor formation ability in vitro. We observed that tumor colonies were markedly smaller than those formed by wild-type tumor cells and mock cells (Fig. 2c). In addition, the statistical analysis confirmed that the number of colonies formed by the two tumor cells types was greatly reduced by *CANT1* overexpression in vitro (Fig. 2d).

To examine the ability of *CANT1* to suppress tumor formation in vivo, we inoculated *CANT1*-overexpressing Weri-Rb1 cells and mock cells into the posterior segments of the eyes of nude mice. Extraocular tissue was removed, and the tumor-bearing eye mass was measured. As expected, the eye mass weight in the *CANT1-*overexpressing group was reduced by 48% (*n* = 7, *P < 0.05; Fig. 2e, Supplementary Fig. S2A), and the eye mass exhibited a marked reduction in size (Fig. 2f). These results demonstrate that the lncRNA *CANT1* serves as a tumor suppressor that modulates tumor formation in RB.

### *CANT1* modulates PI3Kγ/Akt signaling in RB

**Fig. 3:**
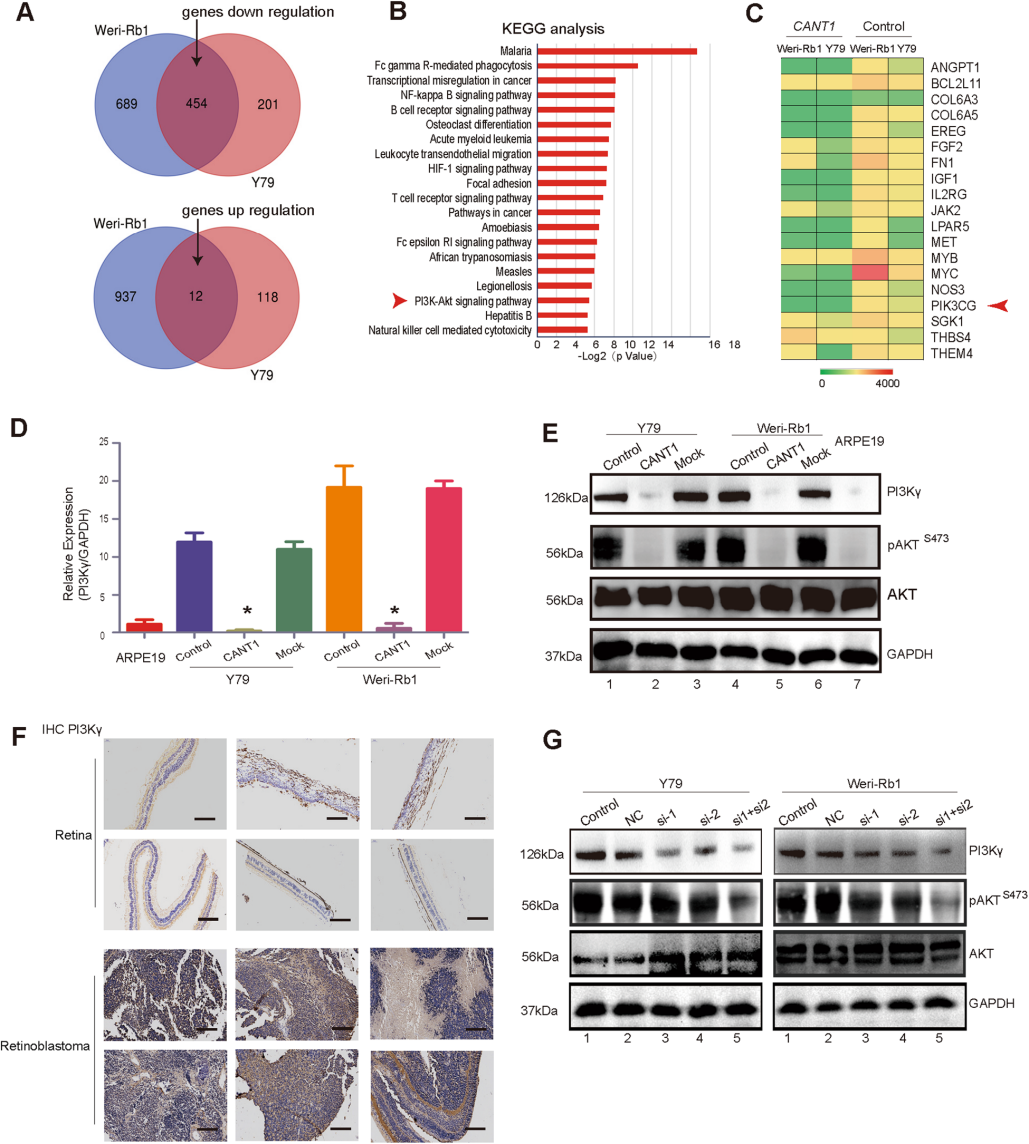
Regulatory targets of *CANT1* lncRNA in RB. **a** The overlapping genes presenting differential expression between the two RB cell lines are shown. Twelve genes were co-upregulated, and 454 genes were co-downregulated (fold change ≥ 1.5, FDR < 0.05). **b** KEGG analysis for all genes with differential expression. **c** Heatmap of genes in the PI3K/Akt signaling pathway. **d** Real-time PCR was performed to measure the *PI3Kγ* mRNA level in tumor and normal cells. All of the data are presented as the mean ± SEM. *P < 0.05: compared with the control and mock. **e** Western blot showing the protein levels of PI3Kγ, p-AKT, and pan-AKT in tumor and normal cells. GAPDH was used as internal control. **f** Immunohistochemical staining of PI3Kγ in RB and normal tissues. PI3Kγ expression in tumor sections was higher than normal tissues. Scale bar: 200 μm. **g** Two siRNAs were used to silence PI3Kγ, as examined by Western blot.

To elucidate the mechanism underlying the suppressive role of *CANT1* in RB expansion, we employed RNA transcriptome-sequencing (GEO Accession number: GSE141327) to analyze *CANT1*-overexpressing cells and control Y79 and Weri-Rb1 cells. We found differentially expressed genes (fold change ≥ 1.5, false discovery rate < 0.05) in both cell lines, with 12 genes upregulated and 454 genes downregulated (Fig. 3a). The Gene Ontology (GO) analysis showed that the most significantly overrepresented biological processes included pathways involved in oxygen transport, regulation of cell population proliferation, as well as signal transduction (Supplementary Fig. S3A). The Kyoto Encyclopedia of Genes and Genomes (KEGG) analysis demonstrated that several pathways, including the NF-κB signaling pathway and PI3K/Akt signaling pathways, were altered (Fig. 3b). Then, we focused on the differentially expressed genes in PI3K/Akt signaling pathway (Fig. 3c) and examined the expression of *PI3Kγ* (also called *PIK3CG*, gene ID: 5294; one of the most important genes in PI3K/Akt signaling) in tumor cells and in normal ARPE19 cells. The RNA-sequence analysis showed that there was almost no transcription at the *PI3Kγ* locus in *CANT1*-overexpressing RB cells (Supplementary Fig. S3B). As expected, at the mRNA level, *PI3Kγ* expression was upregulated more than 10-fold in Y79 cells and approximately 20-fold in Weri-Rb1 cells, compared with that in normal ARPE19 cells (Fig. 3d). The Western blot data confirmed that the protein expression of *PI3Kγ* in RB tumor cells was markedly increased compared to that of normal ARPE19 cells, which displayed extremely weak *PI3Kγ* expression (Fig. 3e, lane1, lane 4, and lane 7). However, in *CANT1*-overexpressing RB cells, both mRNA (Fig. 3d) and protein (Fig. 3e, lane 2 and lane 5) levels of *PI3Kγ* were significantly reduced. We also examined the activation of Akt, downstream of PI3Kγ. The Akt phosphorylation level decreased in accordance with the *PI3Kγ* expression level. An IHC staining was then performed to detect PI3Kγ protein expression in RB tissues compared with normal retina. The results clearly showed that PI3Kγ protein was remarkably increased in the human RB tissues (Fig. 3f). The staining of PI3Kγ in the mouse Weri-Rb1-mock eyes was also darker than that in the Weri-Rb1-*CANT1* eyes obtained from the mouse experiments (Supplementary Fig. S3C). Taken together, these data demonstrate that *PI3Kγ* may act as a potential target of the lncRNA *CANT1*.

### PI3Kγ is required for RB tumorigenesis

To further investigate the role of *PI3Kγ* in RB, we used the classic RNAi method to silence *PI3Kγ* expression in RB cells. Three siRNAs (si*PI3Kγ*-1, si*PI3Kγ*-2, and si*PI3Kγ*-3) were designed to test the efficiency of silencing. The results showed that both si*PI3Kγ*-1 and si*PI3Kγ*-2 worked and an si*PI3Kγ*-mix that combined si*PI3Kγ*-1 and si*PI3Kγ*-2 was more efficient than the two siRNAs alone in knocking down *PI3Kγ* expression at the mRNA transcript level (Supplementary Fig. S4A, B). si*PI3Kγ*-1, si*PI3Kγ*-2, and si*PI3Kγ*-mix were able to knock down the PI3Kγ protein and silence PI3K/Akt signaling in the two RB cell lines (Fig. 3g). Intriguingly, lncRNA *CANT1* expression was not notably changed when *PI3Kγ* was depleted (Supplementary Fig. S4C, D). These data support our hypothesis that diminished *PI3Kγ* expression is triggered by *CANT1* upregulation and *PI3Kγ* acts as a regulatory target of lncRNA *CANT1*.

**Fig. 4:**
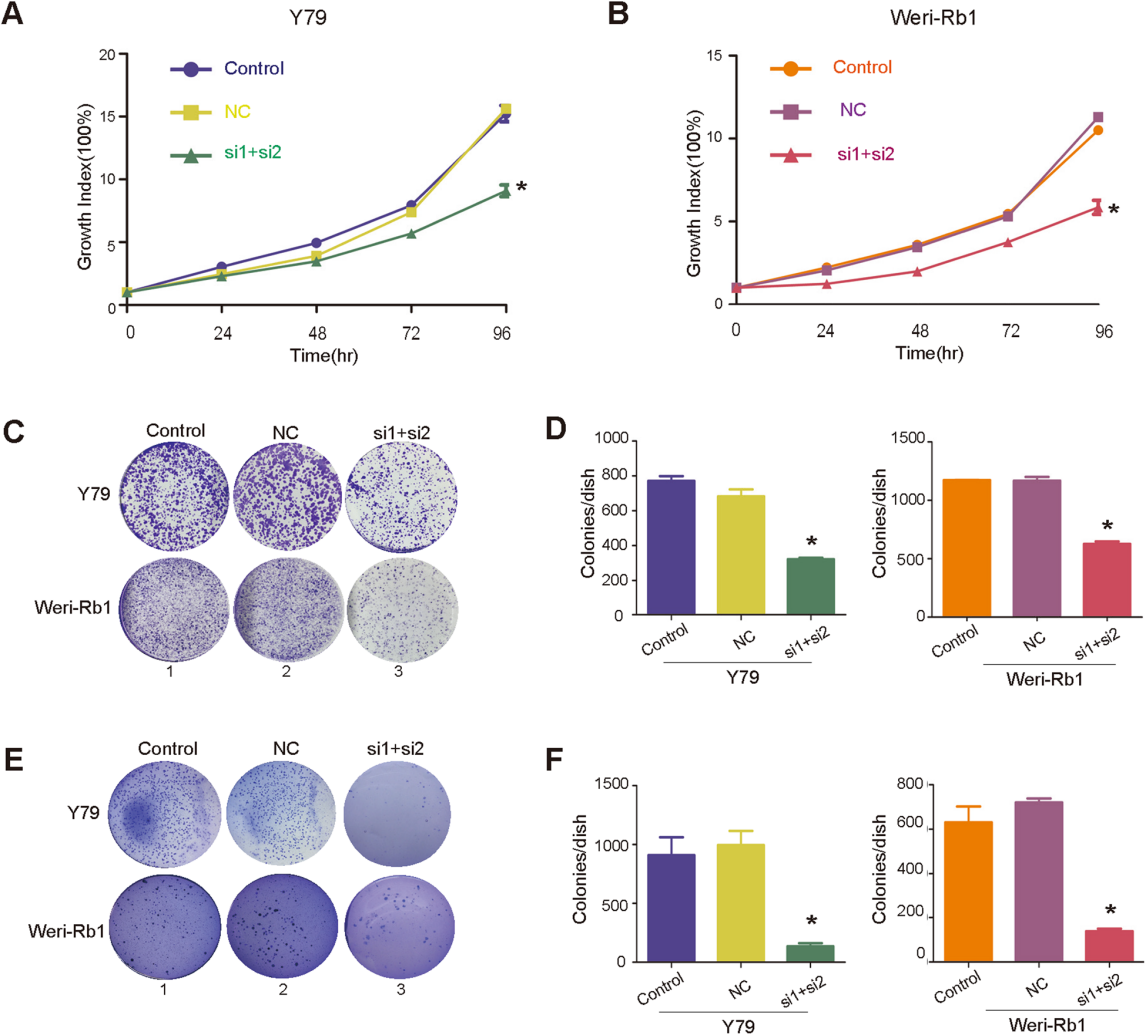
Oncogenic role of *PI3Kγ* in RB. **a**, **b** CCK8 assay showed that the cell growth rate decreased after the silencing of *PI3Kγ*. All of the data are presented as the mean ± SEM. *P < 0.05: compared with the control and NC. **c** Colony formation assay was used to assess the cell growth rate of tumor cells treated with siRNA. **d** Small colonies on each plate were counted. All of the data are presented as the mean ± SEM. *P < 0.05: compared with control. **e** A soft agar assay was performed to estimate the in vitro tumor formation ability of Y79 and Weri-Rb1 cells treated with siRNA. **f** Quantification of visible colonies on each plate. All of the experiments were performed in triplicate, and the number of colonies formed is shown as the mean ± SEM. *P < 0.05.

To further define the role of *PI3Kγ* in tumor formation, we chose si*PI3Kγ*-mix for subsequent experiments. In the CCK8 assay, the proliferation of *PI3Kγ*-knockdown RB cells was found to be significantly decreased (Fig. 4a, b). A colony quantification assay showed that the number of colonies was markedly reduced by siRNA treatment in Y79 and Weri-Rb1 cells (Fig. 4c, d). Furthermore, through a soft agar assay, we found that the in vitro colony formation ability of RB cells was significantly reduced by *PI3Kγ* inactivation (Fig. 4e, f). These data show that *PI3Kγ* may act as an oncogene in RB progression and its attenuation may mediate the antitumor effect of *CANT1* in RB tumorigenesis.

### *CANT1* directly binds to the *PI3Kγ* promoter

**Fig. 5:**
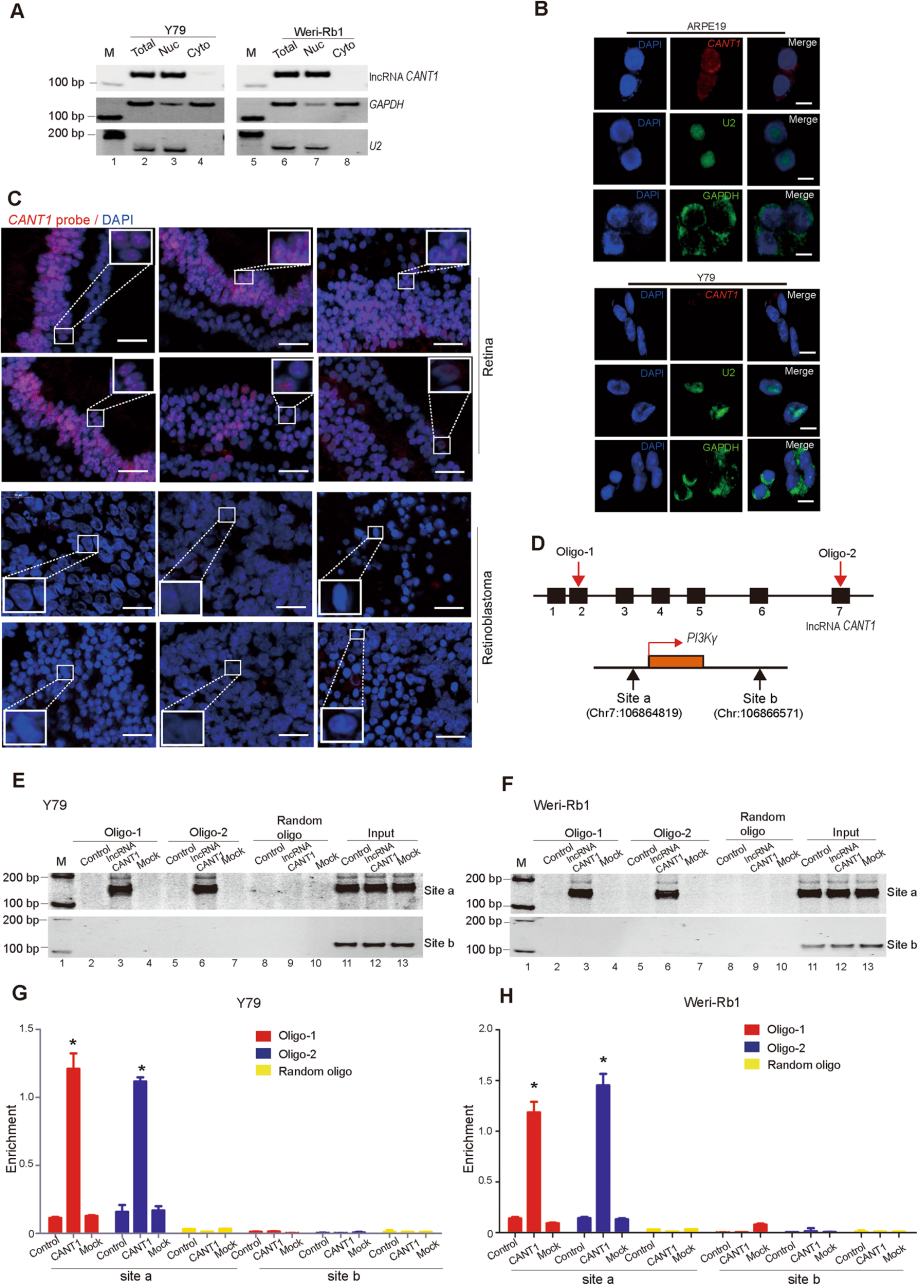
*CANT1* binds to the promoter of its targets. **a** The location of mature *CANT1*. *CANT1* is mainly located in the nucleus. *U2* RNA was used as a positive control for nuclear RNA, and *GAPDH* served as a positive control for the cytoplasmic RNA. **b** Representative RNA FISH images showed that the *CANT1* signal overlapped with DAPI staining in ARPE19 cells. The scale bars represent 10 μm. **c** RNA-FISH was performed with *CANT1* oligos on clinical retinoblastoma samples and normal control samples. Scale bar: 20 μm. **d** Schematic diagram of lncRNA *CANT1* and the *PI3Kγ* promoter region. *CANT1* oligo-1 and oligo-2 indicate the biotinylated antisense oligonucleotides targeting lncRNA *CANT1*. Random oligo indicates the scrambled oligonucleotide used as a negative control in the ChOP assay. **e**, **f** PCR examination of the binding of *CANT1* to the *PI3Kγ* promoter in the ChOP assay. Site a: *CANT1* interacts with the *PI3Kγ* promoter. Site b: a negative control locus. Input: Total RNA was reverse transcribed before incubation with labeled *CANT1* fragments and amplified with primers for site a and site b. **g**, **h** Quantification of the binding of *CANT1* to the *PI3Kγ* promoter in the ChOP assay by real-time qPCR. The data are presented as mean ± SEM. *P < 0.05.

Using U2 snRNA as a positive control for nuclear fraction and GAPDH as a control for the cytosolic fraction, we found that *CANT1* was located mainly in the nucleus in *CANT1*-overexpressing Y79 and Weri-Rb1 cells (Fig. 5a). RNA fluorescence in situ hybridization (RNA FISH) further confirmed that *CANT1* was mainly distributed in the nucleus (Fig. 5b). In addition, RNA FISH demonstrated that *CANT1* RNA was abundant in the cell nucleus of human retina tissues, whereas very weak signals were observed in human retinoblastoma tissues (Fig. 5c). These data suggest that *CANT1* is a nuclear lncRNA in RB cells and might guide *PI3Kγ* regulation via a chromosome-related mechanism. To explore the mechanism by which *CANT1* regulates the expression of *PI3Kγ*, we used a classical ChOP assay. We designed two biotin-labeled, short oligonucleotides aligned with *CANT1* (Fig. 5d, upper). Site a (300 bp upstream of the *PI3Kγ* TSS) was used to detect the promoter region of *PI3Kγ* and site b was used as nonspecific promoter region (Fig. 5d, bottom). After pull-down, we found that *CANT1* bound strongly to the *PI3Kγ* promoter in two *CANT1*-overexpressing cell lines (Fig. 5e, f, lane 3 and lane 6), whereas this DNA–RNA interaction was not observed in the control or mock-transfected cells (Fig. 5e, f, lane 2, lane 4, lane 5, and lane 7). We also used a random oligonucleotide as a negative control because it did not bind to the *PI3Kγ* promoter in either cell line (Fig. 5e, f, lanes 8–10). In addition, to further validate the binding of *CANT1* to the *PI3Kγ* promoter, we performed an RT-qPCR analysis to quantitate the enrichment of *CANT1* at the *PI3Kγ* promoter. The analysis showed that *CANT1* binding to the promoter of *PI3Kγ* was increased compared with that in the controls and mocks (Fig. 5g, h). Taken together, these results demonstrate that *CANT1* might regulate *PI3Kγ* expression by directly binding to key DNA regulatory regions of its promoter.

### *CANT1* modulates *PI3Kγ* expression by abolishing histone H3K4 methylation

**Fig. 6:**
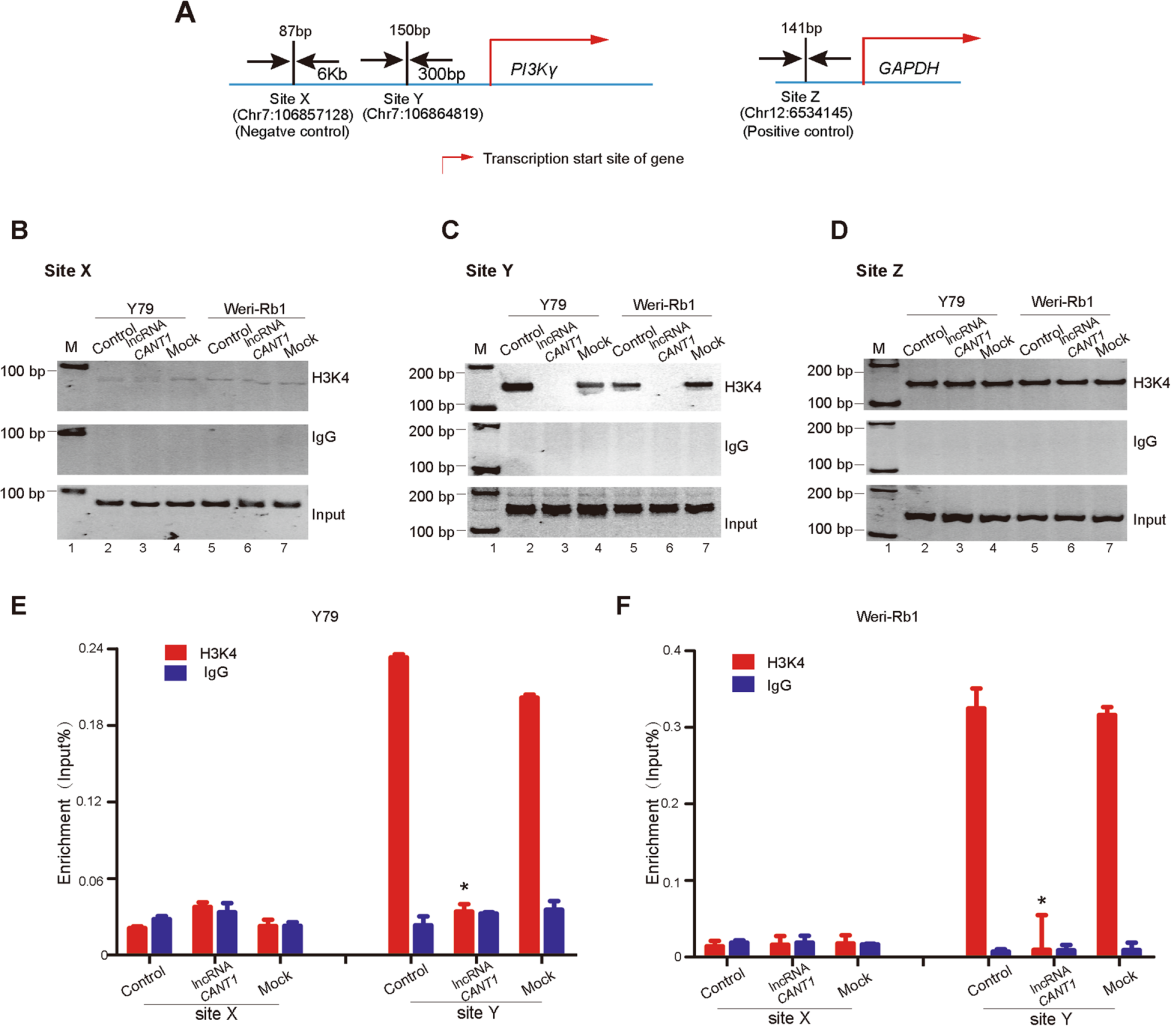
*CANT1* modulates *PI3Kγ* transcription by abolishing H3K4 methylation. **a** Schematic diagram of the *PI3Kγ* and the *GAPDH* promoter regions. Arrow: transcriptional direction; sites X–Z: different sites used in this assay. **b**–**d** PCR examination of histone H3K4 trimethylation changes in the *PI3Kγ* promoter (**c**) and *GAPDH* promoter (**d**) upon *CANT1* overexpression in Y79 and Weri-Rb1 cells. IgG was used as a negative control. Input: total RNA was reverse transcribed before incubation and amplified with primers; M: marker. **e**, **f** Real-time qPCR examination of histone H3K4 trimethylation changes in the *PI3Kγ* promoter. Site X is 6 kb upstream of the *PI3Kγ* TSS. All data are presented as the means ± SEM. *P < 0.05: compared with the control and mock.

Next, we explored whether epigenetic modifications were altered and whether the histone methylation status was changed at the promoter of the *PI3Kγ* gene after *CANT1* overexpression. Site X was 6 kb upstream of the *PI3Kγ* transcription start site (TSS), site Y was 300 bp upstream of the *PI3Kγ* TSS, and site Z was the promoter of *GAPDH* (Fig. 6a). Through a ChIP assay, we found that the H3K4 trimethylation status at the *PI3Kγ* promoter was significantly decreased after *CANT1* overexpression (Fig. 6c). The H3K4 trimethylation status at 6 kb upstream of the *PI3Kγ* TSS (site X) was minimal, whether *CANT1* was overexpressed or not (Fig. 6b). H3K4 modification at the *GAPDH* promoter (site Z) was used as a positive control because *GAPDH* expression was stable in all cell lines (Fig. 6d). The above results were further confirmed by a quantitative ChIP-PCR assay (Fig. 6e, f). Furthermore, we found that there was no significant change in H3K4me3 expression after *CANT1* overexpression (Supplementary Fig. S4E), indicating that *CANT1* itself does not regulate the activity of H3K4 methyltransferase in cell lines and likely regulates H3K4me3 of the *PI3Kγ* promoter by modulating the binding of H3K4 methyltransferase to the target regions of the genome.

### *CANT1* competes with hSET1 methyltransferase *in vitro*

**Fig. 7:**
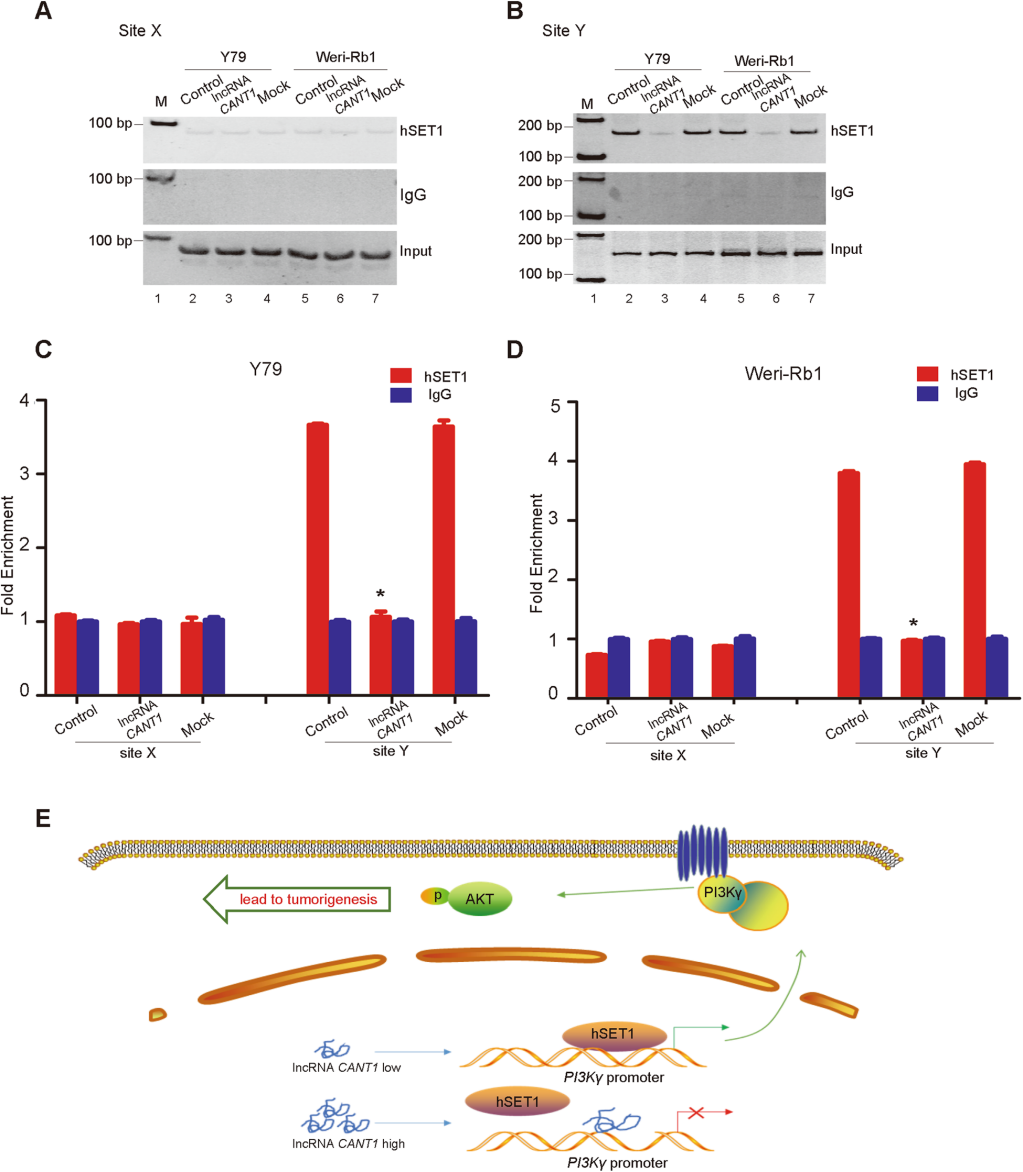
*CANT1* competes with hSET1 at the *PI3Kγ* promoter in vitro. **a**, **b** ChIP assays demonstrated that *CANT1* blocks the recruitment of hSET1 to the *PI3Kγ* promoter. IgG was used as a negative control. Sites X, Y: ChIP detection sites. Input: total RNA was reverse transcribed before incubation and amplified with primers. M: marker. **c**, **d** Real-time qPCR examination of hSET1 changes in the *PI3Kγ* promoter. All data are presented as the means ± SEM. *P < 0.05: compared with the control and mock. **e** Model of *CANT1* regulation in tumorigenesis. In parent cancer cells, *CANT1* lncRNA is inactivated and the hSET1 methyltransferase freely modifies the *PI3Kγ* promoter, providing histone H3K4 methylation to induce *PI3Kγ* expression; however, in *CANT1*-overexpressing cells, *CANT1* occupies the *PI3Kγ* promoter and blocks the hSET1 interaction with the *PI3Kγ* promoter. Then, free hSET1 fails to methylate the *PI3Kγ* promoter and silences *PI3Kγ* expression, thus decreasing PI3K/Akt signaling and inhibiting tumor growth.

Because hSET and MLL1–4 are H3K4 methyltransferases, we sought to identify which participates in the above process. We used a ChIP assay and found that hSET1 bound to the nearby *PI3Kγ* promoter in control and mock cells (Fig. 7b, lane 2, lane 4, lane 5, and lane 7), but the recruitment of hSET1 to the *PI3Kγ* promoter was markedly inhibited in the two *CANT1-*overexpressing cell lines (Fig. 7b, lane 3 and lane 6). We used negative ChIP Site X (6 kb upstream of the *PI3Kγ* TSS) to exclude nonspecific interactions. As expected, hSET1 binding to the *PI3Kγ* promoter could not be measured regardless of the *CANT1* expression status at site X (Fig. 7a). Similarly, the ChIP-qPCR data were consistent with these data (Fig. 7c, d). We also performed an RNA IP assay and found that *CANT1* does not bind to the hSET1 protein. Taken together, these results demonstrate that *CANT1* blocks the hSET1 methyltransferase from binding to the *PI3Kγ* promoter.

## Discussion

Considering that more than 80% of the genome is transcribed and that the majority of the transcription across the genome is non-coding, the chances of identifying a functional potential noncoding RNA are high^27^. A set of characterization studies has identified the critical roles played by long noncoding RNAs in gene expression regulation, cytoplasmic or nuclear complexes scaffolding, and pairing with other RNAs^3,28,29^. However, the functions of the vast majority of these transcripts remain unknown and deserve further investigation. Herein, we revealed that a novel inactivated lncRNA, *CANT1*, at chromosome 6p22.3 modulates RB tumorigenesis through the epigenetic activation of *PI3Kγ* expression, thus enhancing PI3K/Akt signaling and accelerating tumor progression (Fig. 7e).

It has been shown that chromosome 6p22.3 is a tumor susceptibility locus that impacts tumor initiation and progression^30,31^. Ensemble annotation predicted six *CASC15* lncRNA isoforms, two of which (*CASC15-003* and *CASC15-004*) are widely expressed in neuroblastoma, predicting an improved clinical outcome with increased expression^32^. Otherwise, the *CASC15* isoform exerts oncogenic functions in the cutaneous melanoma progression^33^. We previously reported that lncRNA *CANT1* triggers a *CANT1-JPX/FTX-XIST* long noncoding pathway to suppress uveal melanoma progression^15^. Due to the etiology of eye neoplasms differing markedly from cutaneous melanomas and neuroblastomas, it is not surprising that the *CANT1* isoform exists in RB and acts as a tumor suppressor that affects the characteristics of RB.

Studies have shown that class 1 phosphoinositide 3-kinases(PI3Ks), consisting of *PI3Kα*, *PI3Kβ*, *PI3Kδ*, and *PI3Kγ*, are a family of dual-specificity lipid and protein kinases that phosphorylate the inositol ring of phosphoinositides and then activate Akt, a serine/threonine kinase that directly phosphorylates a wide variety of targets participating in cell growth and survival^34,35^. *PI3Kγ* is a gene that plays different roles in various human malignancies. *PI3Kγ* is frequently deleted in myeloid malignancies and is evaluated as a candidate myeloid tumor suppressor gene^36^. However, in T-cell acute lymphoblastic leukemia, *PI3Kγ* can alone support leukemogenesis in the absence of PTEN phosphatase tumor suppressor function and serves as a nonclassical oncogene^37^. Others have reported that *PI3Kγ* may represent a suitable molecular target for therapeutic intervention in Kaposi’s sarcoma and claudin-low breast cancer^38–40^. Study has shown that down-regulation of *PI3Kγ* expression and hypermethylation at CpG sites of the promoter regions were also detected in primary colon cancers^41^. It is reasonable for us to explore the methylation status of *PI3Kγ* promoter region. In our study, we examined histone H3K4me3, H3K9me3 and H3K27me3 levels after lncRNA *CANT1* overexpression. We found that histone H3K4me3 level was significantly changed after lncRNA *CANT1* overexpression in this locus. Constitutive activation of PI3K/Akt signaling contributes to human neoplasias including RB^42^. In our study, *CANT1* serves as a tumor suppressive lncRNA by modulating its downstream targets. By KEGG analysis, there are several signaling pathways predicted to be regulated by *CANT1* (Fig. 3b). *CANT1* inhibits RB progression depending in part on PI3K/Akt signaling attenuation, in which *PI3Kγ* is a key regulatory element.

It has been reported that the functional mechanisms of lncRNAs are diverse; here, we only considered lncRNA transcriptional regulation at the chromatin level because *CANT1* was mainly localized in the nucleus. The activities of lncRNAs can affect neighboring intrachromosomal genes in cis or target genes on different chromosomes in trans. LncRNAs can regulate distant genes by serving as a scaffold for the assembly of multiple regulatory molecules at a single locus, or can also modulate chromatin structures by recruiting chromatin-modifying enzymes, resulting in expression or repression of a large number of genes^3,13,28^. LncRNA *NBAT1* (also called *CASC14*), the expression of which is decreased in neuroblastoma, functionally interacts with PRC2 member EZH2 and controls the expression of target genes via the loss of H3K27me3 from their promoter regions, resulting in neuroblastoma development^43^. However, lncRNAs can also serve as insulators to block regulatory factors from binding to DNA elements such as promoters involved in transcription. We previously reported that linc-*ROR* may function as a decoy oncoRNA that frees the histone methyltransferase G9A from binding to its target gene, promoting tumorigenesis^13^. In this report, we show that *CANT1* is silenced in retinoblastoma and thus hSET1 methyltransferase is recruited to the *PI3Kγ* promoter, promoting its H3K4 trimethylation and activating *PI3Kγ* expression. When *CANT1* was overexpressed in RB cells, it occupied the *PI3Kγ* promoter and blocked hSET1 methyltransferase; consequently, H3K4me3 was lost from *PI3Kγ* promoter region.

It should be noted that RB is the most common intraocular tumor in childhood and the genetic basis of its occurrence and progression has been researched for decades. The classical hypothesis is that biallelic loss of the *RB1* gene results in the defective formation of the RB protein and triggers tumor initiation^16^. However, the mechanisms behind this rapid evolution of the tumor go far beyond *RB1* inactivation. Further genetic or epigenetic alterations, such as *MYCN* amplification, inactivating mutations of *BCL-6* co-repressor (*BCOR*), the aberrant expression of *SYK*, DNA copy number gains of *KIF14* and noncoding RNAs, likely contribute to the malignant transformation^14,16,18,19,21,44^. In addition, we previously reported for the first time that lncRNA *GAU1* is a novel oncoRNA that promotes RB tumorigenesis^14^. In this study, through the genomic analysis of our previous profiling data, we identified that lncRNA *CANT1* expression is markedly downregulated in RB tissues compared to that in normal tissues. Low expression levels of *CANT1* correlated with some clinicopathological factors, such as tumor growth rate and increased tumor size, suggesting that *CANT1* might be a potential therapeutic target and prognostic indicator of RB.

In aggregate, our data suggest a novel mechanism in which lncRNA *CANT1* serves as a tumor suppressor and outline a new pattern of histone modification in RB tumorigenesis. It is possible that *CANT1* may block target gene expression by binding to DNA elements. Understanding the various roles of lncRNAs in tumor progression enables us to better explain the disease phenotype, improve the treatment regimens and provide reliable lncRNA-based prognostic markers.

## Supplementary information

clean version of supplementary figure legends

Supplementary table 1

Supplementary table 2

supplementary figure 1

supplementary figure 2

supplementary figure 3

supplementary figure 4
