# Supplementary material for: LncRNA CANT1 suppresses retinoblastoma progression by repellinghistone methyltransferase in PI3Kγ promoter
Source: Cell Death Dis. 2020 May 4;11(5):306. doi: 10.1038/s41419-020-2524-y (PMC7198571; doi:10.1038/s41419-020-2524-y)
Supplement: Supplementary file 1 — clean version of supplementary figure legends [file 41419_2020_2524_MOESM1_ESM.docx]

**Supplementary files**

**LncRNA *CANT1* suppresses retinoblastoma progression by repelling**

**histone methyltransferase in *PI3Kγ* promoter**

Hongyan Ni^1,2*^, Peiwei Chai^1,2*^, Jie Yu^1,2*^, Yue Xing^1,2*^, Shaoyun Wang^1,2^, Jiayan Fan^1,2^, Shengfang Ge^1,2^, Yefei Wang^1,2#^, Renbing Jia^1,2#^, Xianqun Fan^1,2#^

**Correspondence:** Yefei Wang (paper34@163.com), Renbing Jia (renbingjia@sjtu.edu.cn), or Xianqun Fan (fanxq@sjtu.edu.cn)

**Affiliations and contact information:**

^1^Department of Ophthalmology, Ninth People’s Hospital, Shanghai JiaoTong University School of Medicine, Shanghai, China, 200011.

^2^Shanghai Key Laboratory of Orbital Diseases and Ocular Oncology, Shanghai, China, 200011.

Tel: +86 21 63135606; Fax: +86 21 63135606.

^*^These authors contributed equally to this work: Hongyan Ni, Peiwei Chai, Jie Yu, Yue Xing

**Keywords:** *CANT1*; PI3Kγ; hSET1; Tumorigenesis

**Running title:** Aberration lncRNA expression induces tumorigenesis

Supplemental information includes 4 figures and 2 tables.

**Supplementary figure legends**

**Figure S1.**

**(A)** Genomic structure of *CANT1*. The black and grey rectangles indicate the exons of *CASC15* and *CANT1*, respectively. The arrows show the specific primers for *CASC15* and *CANT1*.

**Figure S2.**

**(A)** General photograph of orthotopic xenografts 40 days after implantation via the injection of Weri-Rb1 cells into the vitreous space with or without *CANT1* overexpression; n = 7. T: tumor; N: normal.

**Figure S3.**

**(A)** RNA-sequence analysis results. Enriched GO terms of the potential target mRNAs according to the biological process. **(B)** RNA-sequence results showing the transcriptome in parent RB cells and *CANT1* overexpressing RB cells. Blue chart: the exons of *PI3Kγ*. (**C**) Representative images of PI3Kγ immunohistochemical staining in mouse RB eyes and normal mouse retina. scale bar: 15 μm.

**Figure S4.**

**(A and B)** Real-time qPCR showing *PI3Kγ* mRNA levels in Y79 and Weri-Rb1 cells treated with siRNA. Control: wild-type tumor cells; all data are presented as the means ± SEM. *P <0.05: compared with the control. **(C and D)** Real-time qPCR was used to measure the lncRNA *CANT1* level in tumor cells upon *PI3Kγ* knockdown. Control: Wild type tumor cells; All data are presented as the means ± SEM. *P <0.05: compared with the control. **(E)** The global protein level of H3K4me3 and H3K4me change in RB cells after *CANT1* overexpression.
