# Supplementary material for: LncRNA CANT1 suppresses retinoblastoma progression by repellinghistone methyltransferase in PI3Kγ promoter
Source: Cell Death Dis. 2020 May 4;11(5):306. doi: 10.1038/s41419-020-2524-y (PMC7198571; doi:10.1038/s41419-020-2524-y)
Supplement: Supplementary file 2 — Supplementary table 1 [file 41419_2020_2524_MOESM2_ESM.docx]

**Supplementary Table 1 Primers, siRNAs and oligonucleotides sequences used in this study.**

| Primer name | Sequences (5'-3') | Purpose |
| --- | --- | --- |
| GAPDH-F  GAPDH-R  CANT1-F  CANT1-R  CASC15-F  CASC15-R  PI3Kγ-F  PI3Kγ-R  U2-F  U2-R  siPI3Kγ-1  siPI3Kγ-2  siNC  CANT1-oligo-1  CANT1-oligo-2  Random oligo  PI3Kγ-site a-F  PI3Kγ-site a-R  PI3Kγ-site b-F  PI3Kγ-site b-R  PI3Kγ-site X-F  PI3Kγ-site X-R  PI3Kγ-site Y-F  PI3Kγ-site Y-R  GAPDH-site Z-F  GAPDH-site Z-R | AGGTCGGTGTGAACGGATTTG  TGTAGACCATGTAGTTGAGGTCA  GAGCTGAAACTGTGTGAATCATGG  GGAAGGAAGTAAATCTTGGAGTCCT  GAGTACTTCCTAGCACTGACCTCCT  AAGCAACTCCAGATGAATCCAGGAA  GCACACTGGGTCGCATAGG  GTTTCGGGGCTCTTGCATTT  TATCTGATACGTCCTCTATCCGAGG  GTTCCTGGAAGTACTGCAATACCAG  GGTACGAGATCTACGACAA  GAGTACCTGTGGAAGAAGA  ATCCACTACCGTTGTTATAGGTG  GTCCTGCACTTCCATCCGTT  ACTCCCATCTTGCTTGCACA  GCGCTATGAAGAAACTTGGC  GCTAAGGAAGTGAGCTTGCAG  TTAAACTCATTCCCCTCCTGGG  TCCATCCTGTAGCTCCTCCAT  GTCACCCAAGGCTGTACTCT  CATCCAACCTGCATGGGACT  TGCTAACTGTGCTGATCGCT  GCTAAGGAAGTGAGCTTGCAG  TTAAACTCATTCCCCTCCTGGG  AGTCGTTCCCAAAGTCCTCC  GGGGGAAGGGACTGAGATTG | PCR  PCR  PCR  PCR  PCR  PCR  PCR  PCR  PCR  PCR  siRNA  siRNA  siRNA  CHOP  CHOP  CHOP  CHOP  CHOP  CHOP  CHOP  ChIP  ChIP  ChIP  ChIP  ChIP  ChIP |
