# Supplementary material for: LncRNA CANT1 suppresses retinoblastoma progression by repellinghistone methyltransferase in PI3Kγ promoter
Source: Cell Death Dis. 2020 May 4;11(5):306. doi: 10.1038/s41419-020-2524-y (PMC7198571; doi:10.1038/s41419-020-2524-y)
Supplement: Supplementary file 3 — Supplementary table 2 [file 41419_2020_2524_MOESM3_ESM.doc]

| **Supplementary Table 2. The clinical characteristics of retinoblastoma patient cohorts.** | | | | |
| --- | --- | --- | --- | --- |
|  | Features | Retinoblastoma | Unaffected |  |
|  | Numbers | 10 | 5 |  |
|  | Sex |  |  |  |
|  | Male | 6 | 2 |  |
|  | Female | 4 | 3 |  |
|  | Age | 3.7±2.09 | 33.28±3.46 |  |
|  | ICRB group |  |  |  |
|  | A stage | 0 | / |  |
|  | B stage | 0 | / |  |
|  | C stage | 0 | / |  |
|  | D stage | 0 | / |  |
|  | E stage | 10 | / |  |
|  | Laterality |  |  |  |
|  | Unilateral | 4 | / |  |
|  | Bilateral | 6 | / |  |
