# Supplementary figures and images for: LncRNA CANT1 suppresses retinoblastoma progression by repellinghistone methyltransferase in PI3Kγ promoter
Source: Cell Death Dis. 2020 May 4;11(5):306. doi: 10.1038/s41419-020-2524-y (PMC7198571; doi:10.1038/s41419-020-2524-y)

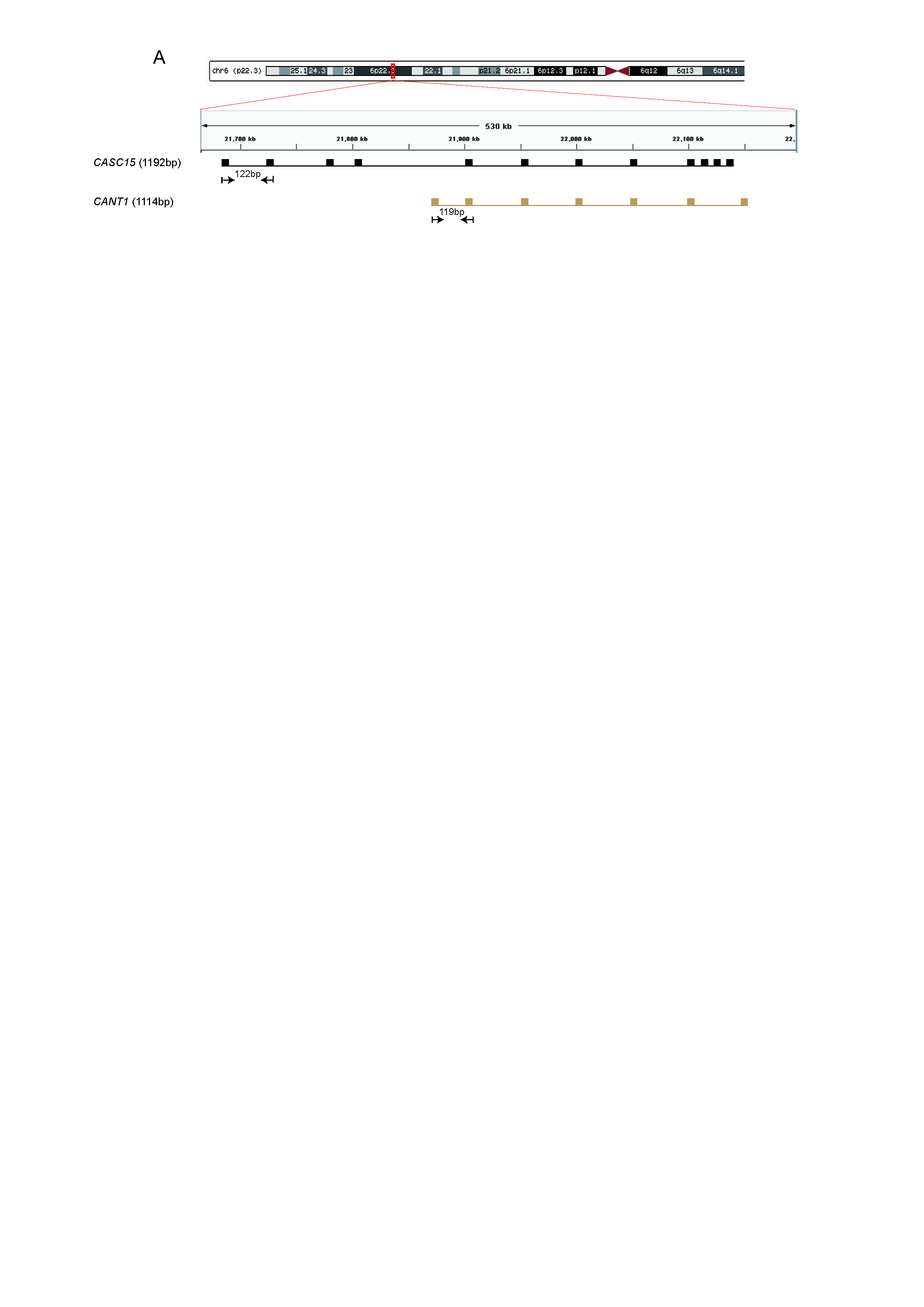

Supplement: Supplementary file 4 — supplementary figure 1 [file 41419_2020_2524_MOESM4_ESM.tif]

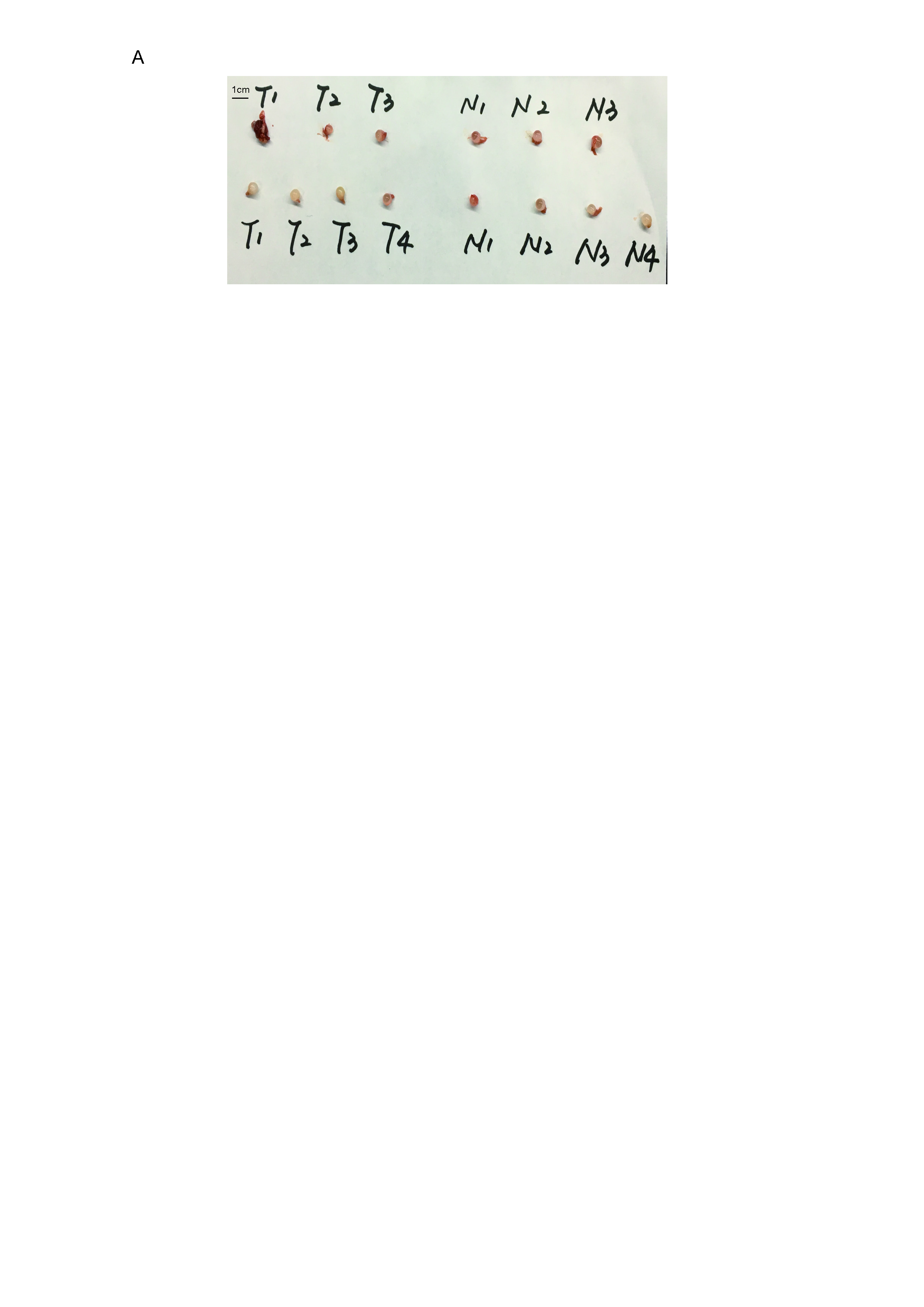

Supplement: Supplementary file 5 — supplementary figure 2 [file 41419_2020_2524_MOESM5_ESM.tif]

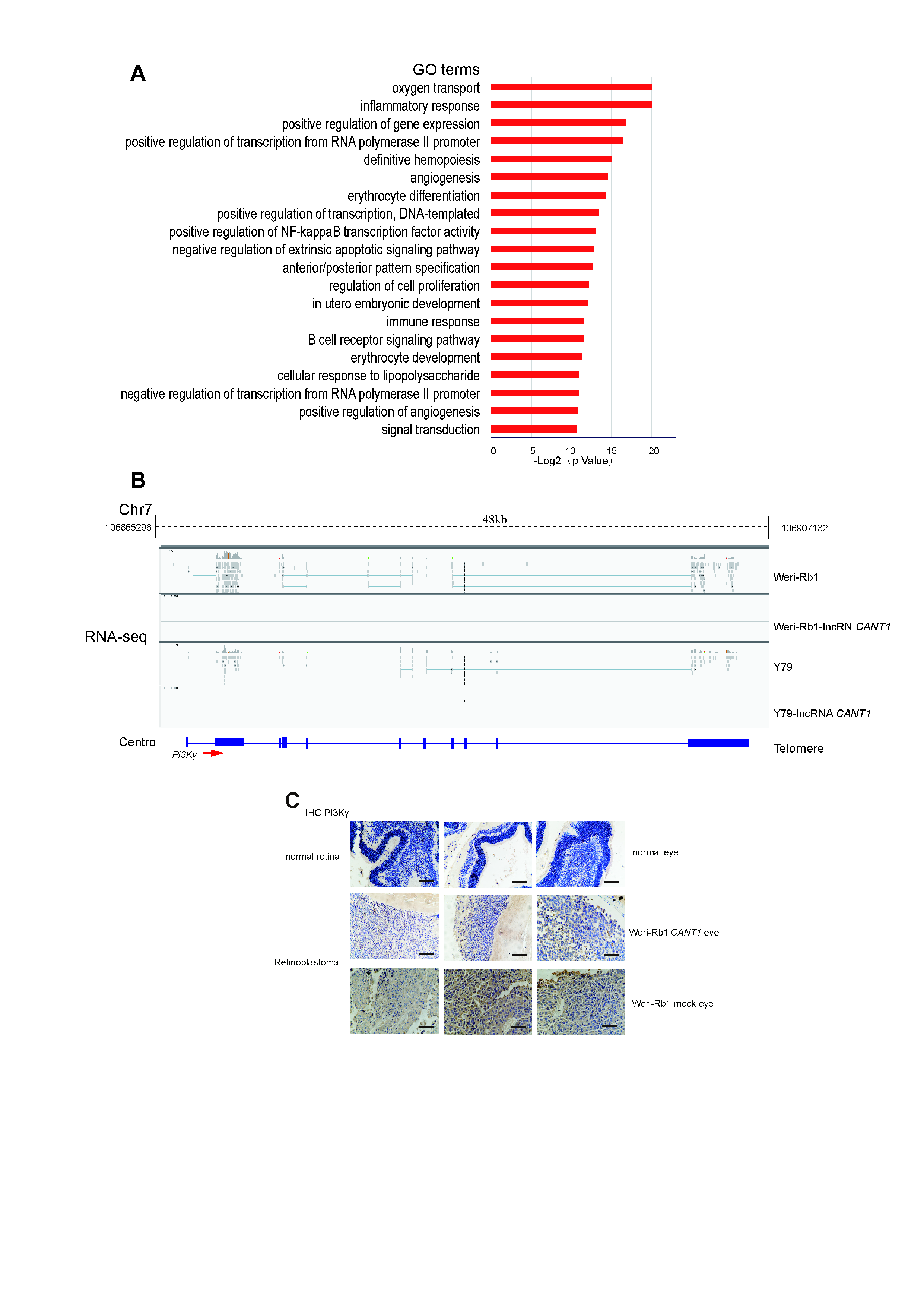

Supplement: Supplementary file 6 — supplementary figure 3 [file 41419_2020_2524_MOESM6_ESM.tif]

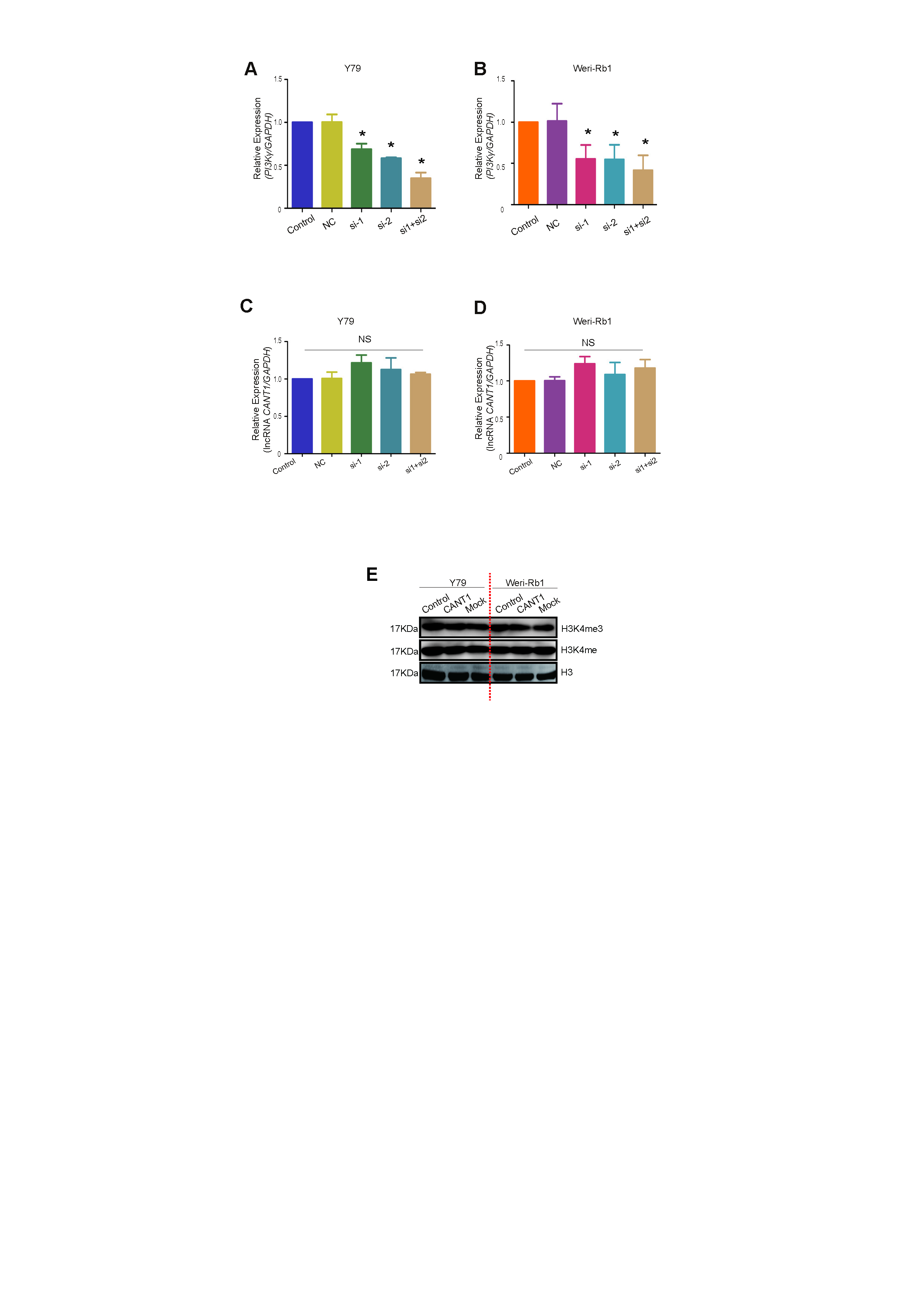

Supplement: Supplementary file 7 — supplementary figure 4 [file 41419_2020_2524_MOESM7_ESM.tif]
